# Effects of a Traditional versus an Alternative Strengthening Exercise Program on Shoulder Pain, Function and Physical Performance in Individuals with Subacromial Shoulder Pain: A Randomized Controlled Trial

**DOI:** 10.3390/sports8040048

**Published:** 2020-04-13

**Authors:** Simon Schedler, Dennis Brueckner, Marco Hagen, Thomas Muehlbauer

**Affiliations:** Division of Movement and Training Sciences/Biomechanics of Sports, University of Duisburg-Essen, 45141 Essen, Germany; simon.schedler@uni-due.de (S.S.); dennis.bruecker@uni-due.de (D.B.); marco.hagen@uni-due.de (M.H.)

**Keywords:** rotator cuff, manual shoulder-training device, shoulder pain, flexibility, maximal strength, strength endurance

## Abstract

A manual shoulder-training device may represent an alternative training device to improve symptoms and function in patients with subacromial shoulder pain by strengthening the external rotators. Thus, we examined the effects of a traditional versus an alternative strengthening exercise program on shoulder pain/function and physical performance in individuals with subacromial shoulder pain. Fifty-six adults with subacromial shoulder pain were randomly assigned to a passive control group (CON; *n* = 20), a traditional training group (TRA; *n* = 19), or an alternative training group (ALT; *n* = 17). Both training groups conducted a progressive home-based strengthening exercise program for the external rotators for eight weeks using elastic bands only (TRA group) or in combination with the shoulder-training device (Schulterhilfe^®^) (ALT group). Pre- and post-training assessment included measures of shoulder pain/function (i.e., shoulder pain and disability index (SPADI)) and physical performance (i.e., shoulder flexibility, maximal isometric strength, and strength endurance). We found significant test × group interactions in most of the investigated variables. Post hoc analyses showed significant training-related improvements for proxies of shoulder pain/function, shoulder flexibility, maximal isometric strength, and strength endurance in favor of the ALT and TRA group in comparison to the CON group. Further, larger and more frequent effects were found for the ALT compared to the TRA group. Measures of shoulder pain/function and physical performance can be significantly improved by both training regimens in individuals with subacromial shoulder pain. However, strength training using elastic bands with the manual shoulder device (ALT group) as compared to elastic bands (TRA group) only was more effective and may thus be a recommendable alternative in order to mitigate subacromial shoulder pain.

## 1. Introduction

Shoulder disorders present one of the most common musculoskeletal, orthopedic disorders in adult humans with estimated incidence rates of 10–15/1000/year [1,2] and a lifetime prevalence of approximately 10% [3]. The majority of people presenting to primary care with shoulder pain are middle-aged adults (age range: 45–65 years) [2]. Subacromial impingement syndrome (SIS) or subacromial shoulder pain is among the most prevalently diagnosed shoulder disorder [1]. It is characterized by a narrowing of the subacromial space, encroachment of surrounding tissues, and tendon pathology [4]. Etiologies are manifold and include subacromial bursitis, rotator cuff tendinopathy, or rotator cuff tears [5]. In most of the cases, it remains unclear whether these result from primarily intrinsic (e.g., time-related degeneration of the tendon) or extrinsic (e.g., compression caused by faulty posture) factors or a combination of both as patients usually show symptoms of both categories once they consult a physician [4].

People affected by subacromial shoulder pain usually suffer from severe shoulder pain [5], restricted flexibility (i.e., range of motion (ROM)) [5], and limited strength [6] of the shoulder muscles especially with respect to internal and external rotation as well as reduced sleep quality [7]. Besides this deterioration in peoples’ perceived quality of life, shoulder disorders also constitute a huge burden for the health and economic sector due to associated sick leave and medical costs [8]. Studies [5,8] have focused on the treatment options for subacromial shoulder pain ranging from conservative therapy such as oral medication with nonsteroidal anti-inflammatory drugs, steroid injections, or physical therapy (PT) [8] to surgery [5]. However, there is no evidence of the superiority of a single intervention, albeit recent systematic reviews with meta-analyses suggest that surgical interventions may not be beneficial compared to conservative treatments [9,10].

In contrast to typical PT sessions, which usually include several strengthening exercises and the use of sophisticated equipment, a single-exercise approach has been suggested and proved to be equally effective as traditional multi-exercise approaches in the short- (three months), middle- (six months), and long-term (twelve months) in patients with rotator cuff tendinopathy [11]. One of the typical or traditional exercises to mitigate subacromial shoulder pain is to strengthen the rotator cuff by performing external rotations with the elbow attached to the torso and flexed to 90° [12]. However, the overall or long-term effectiveness of current treatment options seems to be limited [13].

**Figure 1:**
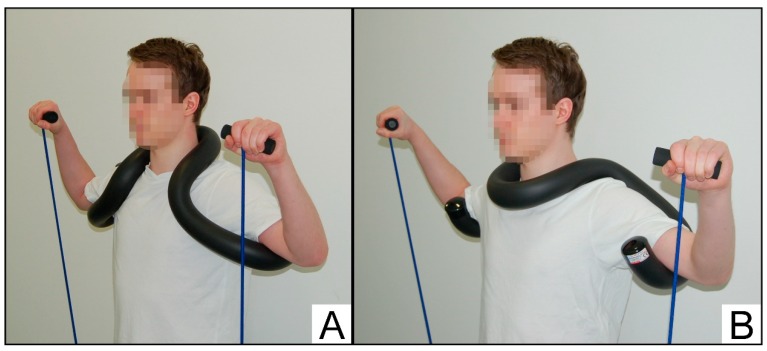
The manual shoulder-training device Schulterhilfe^®^ (AktiFlex Produkte KG, Heusenstamm, Germany) placed around the neck (**A**) or breast (**B**).

Grigereit et al. [14] investigated the effectiveness of a shoulder device aiming to support the arms in abducted position to reduce the activity of the deltoid muscle during shoulder rotation. These authors [14] indeed reported decreased activity of the deltoid muscle when performing external rotations with compared to without wearing the shoulder device and concluded that training with the device might be more effective to explicitly strengthen the external rotators. The succeeding model of the shoulder device (Schulterhilfe^®^, AktiFlex Produkte KG, Heusenstamm, Germany) has been designed to optimize elastic band strength training for the external rotator muscles (Figure 1). The custom-built, synthetic, rubber-covered frame is placed around either the back (Figure 1A) or front (Figure 1B) of the neck, intending to support the arms in abducted position, to reduce intraarticular pressure, and to increase subacromial space. It is hypothesized that elastic band strength training using this isolated alternative (ALT) is advantageous over traditional (TRA) exercises for the external rotators which do not restrict movements in the transverse and frontal planes. However, to the best of our knowledge, no study so far has investigated the effectiveness of a strength training using the shoulder-training device. Thus, the aim of the present study was to investigate the effects of a TRA versus an ALT home-based strengthening exercise program on measures of shoulder pain/function and physical performance in individuals with subacromial shoulder pain. We hypothesized that both exercise conditions will result in improvements but with larger enhancements in the ALT as compared to the TRA training group. 

## 2. Materials and Methods

### 2.1. Participants

Participants were recruited via advertisements in local media (i.e., radio and newspapers) and by publishing a study call on the university website. Inclusion criteria were diagnosed subacromial shoulder pain (e.g., self-report and doctor’s report) with symptoms lasting for at least one year and being 35–65 years of age. People suffering from subacromial shoulder pain for less than a year were excluded from the study to prevent the participation of people with acute or momentary shoulder pain. Further exclusion criteria were other shoulder pathologies (e.g., frozen shoulder) or other known musculoskeletal, neurological, or orthopedic disorders. Persons interested in the study were registered and invited to an informative meeting after which they could decide whether to take part in the study. All potential participants underwent a prescreening regarding their shoulder impairment and its history as well as concerning their shoulder pain/function.

Thereafter, sixty-nine eligible participants were randomly assigned to either a control group (CON) (*n* = 22), a TRA training group (*n* = 23), or an ALT training group (*n* = 24) using Research Randomizer, a program published on a publicly accessible official website (www.randomizer.org). Groups were matched in terms of age, sex, and self-reported shoulder pain/function. Accordingly, no significant differences were observed between groups. Participants’ characteristics are presented in Table 1.

**Figure 2:**
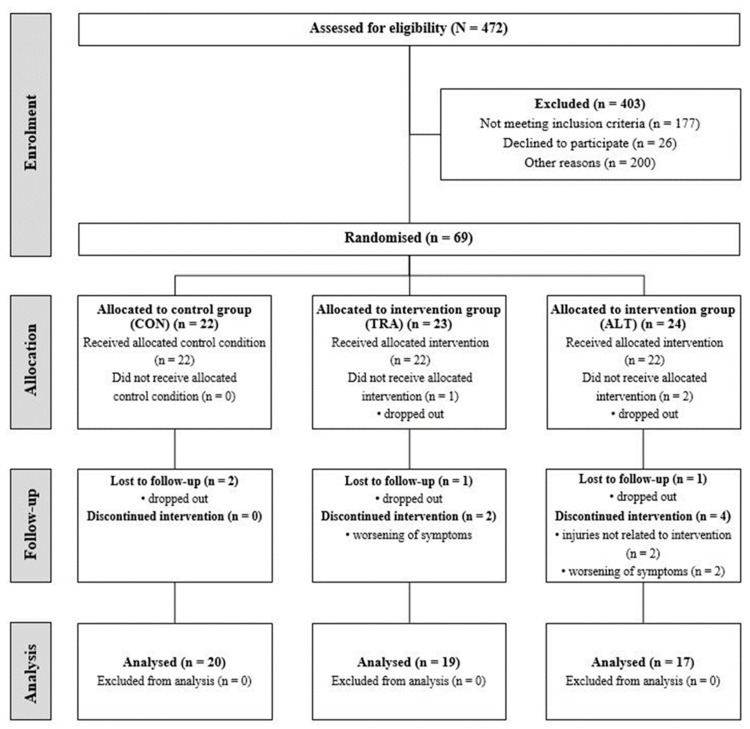
Flowchart of the randomized controlled study design.

Over the course of the study, two participants of the CON, four participants of the TRA, and seven participants of the ALT groups dropped out of the study for various reasons (e.g., injury unrelated to intervention or worsening of symptoms). Figure 2 displays a flowchart of the randomized controlled study design. Participants’ written informed consents were obtained prior to the start of the study. The Human Ethics Committee at the University of Duisburg-Essen, Faculty of Educational Sciences approved the study protocol (TM_11.03.2019), and the study was conducted according to the Declaration of Helsinki [15].

### 2.2. Shoulder Training Programs

**Figure 3:**
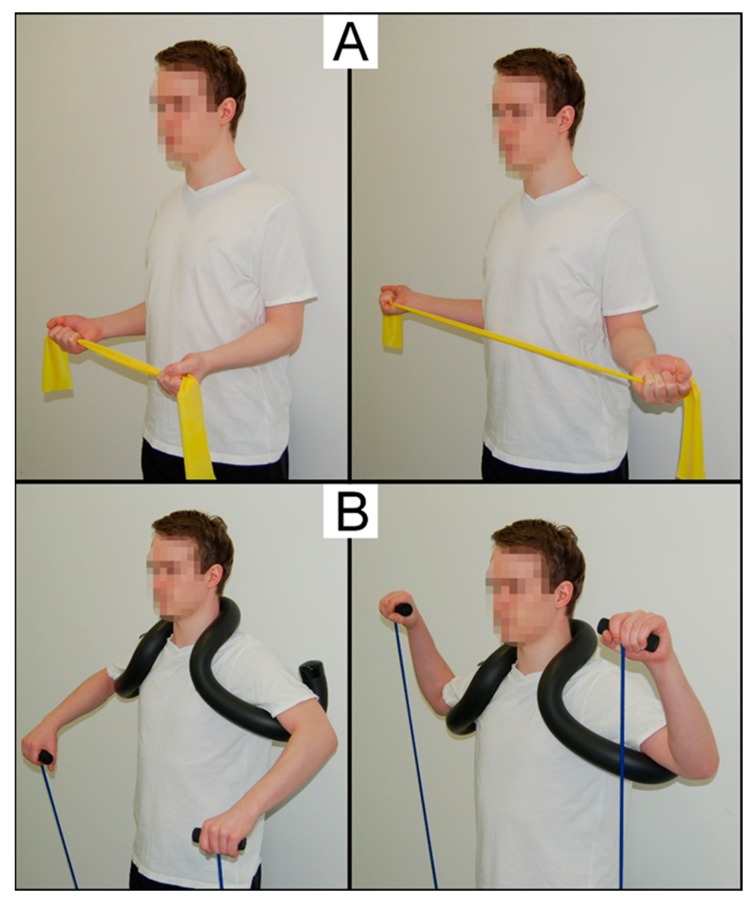
Strengthening exercise for the traditional (**A**) and alternative (**B**) training group.

**Figure 4:**
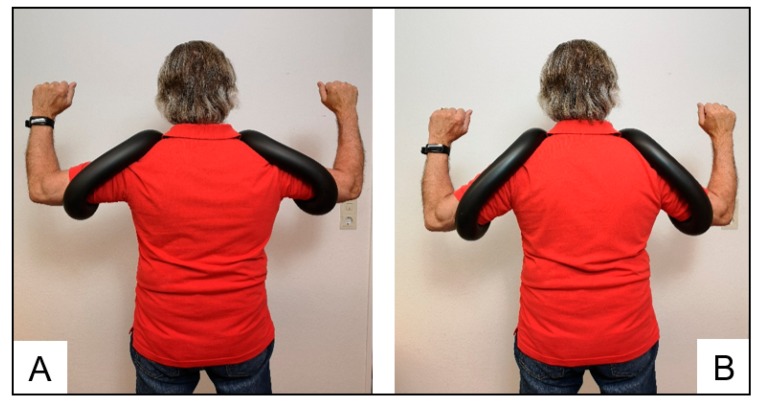
Illustration of the manual shoulder-training device (**A**) “Schulterhilfe^®^ Standard” (i.e., supporting the arms in 90° abduction) and (**B**) “Schulterhilfe^®^ Akut” (i.e., supporting the arms in 45° abduction due to limited flexibility).

The participants of the two training groups conducted a home-based progressive elastic resistance exercise program of the rotator cuff muscles for eight weeks (three sessions/week). Using elastic Therabands^®^ (The Hygenic Corporation, Akron, OH, USA), the TRA group performed external rotations with the upper arm positioned in parallel to the trunk and the elbow joint in approximately 90° (Figure 3A). The ALT group performed external rotations using the manual shoulder-training device, which supported the arms in abducted position, and using appendant elastic bands, which are delivered with the device (Figure 3B). All participants were instructed to perform the movement slowly and controlled, trying to achieve a 2:1-seconds eccentric-concentric ratio. Twenty-three participants in the ALT group used the standard version of the shoulder device (Schulterhilfe^®^ Standard, Figure 4A), supporting the arms in 90° abduction, while one trained with the alternative model (Schulterhilfe^®^ Akut, Figure 4B), supporting the arms in 45° abduction due to limited flexibility. In both programs, one training session included seven sets with a decreasing level of reps (1st set = 18–19 reps to 7th set = 6–7 reps) and 90-seconds rest periods between sets, which were performed after a warm-up consisting of two sets (10–15 reps each). Warm-up exercises were the same as the training exercise but performed without external resistance (e.g., elastic bands). Participants of both training programs were familiarized with the BORG scale during pretest and instructed to perceive a very high level of exertion (17–19 on the BORG scale) in each training set, and this was the most important criterion if the number of repetitions could not be achieved in a certain training set. If participants did not reach the desired level of exhaustion, they were advised to increase the resistance of the elastic band. Progression was achieved by gradually increasing the resistance. Initially, participants were instructed to shorten their elastic band before switching to a band of higher resistance. In both groups, elastic bands were matched for resistance with those used in the ALT group covering a range of approximately 0.3–6.0 kg and with the Therabands^®^ used in the TRA group covering a range of 1.3–2.6 kg at 100% elongation. Participants in the TRA group were also advised to double-grab (i.e., fold) the band in order to increase resistance. The exercise protocol followed the recommendations of the exercise booklet delivered with the shoulder-training device. Each training session lasted approximately 20–25 min.

Additionally, an illustrated exercise booklet was provided for the home-based sessions. All participants of the two exercise groups received their respective training devices and a visual demonstration of the exercise after the pretesting. Further, a training log was given to all participants and they were asked to document each completed training session and the respective stage of progression (band/rope color/shortening). Every fortnight, the quality and quantity of the training as well as the occurrence of pain during the training were controlled by phone calls. All participants (including the CON group) were instructed to maintain their habitual physical activity level and to refrain from all extraneous upper-body resistance training for the duration of the study. Participants of the CON group received the ALT training program when the study was finished.

### 2.3. Testing Procedures

Both the pre- and posttesting was conducted by the same skilled assessors (i.e., graduated sport scientists) in the same laboratory. All participants received standardized verbal instructions and a visual demonstration regarding the testing procedure that included assessment of anthropometric variables, shoulder pain/function, shoulder flexibility, maximal isometric strength (MIS), and strength endurance, which will be described in detail in the following sections. Visual demonstrations of each test were given before participants performed the respective test. This sequence of measurements was the same during the pre- and posttesting. Similarly, repeated measurements performed in a randomized order during pretesting were conducted in the same order during posttesting. Unless otherwise stated, participants were given one practice trial before test trials were recorded. To ensure maximal performance, all participants were requested to finish their respective training program and to abstain from any demanding activities two days prior to pre- and posttesting.

#### 2.3.1. Assessment of Anthropometric Variables

Assessment of anthropometric variables included measures of body height, body mass, and body mass index (BMI). Body height was assessed without shoes to the nearest 0.1 cm using a standardized stadiometer (seca 217, Basel, Switzerland). Further, body mass was measured in light sport clothes and without shoes to the nearest 100 g using an electronic scale (seca 803, Basel, Switzerland). Body mass index was calculated as body mass divided by height squared (kg/m²). McKenna et al. [16] reported excellent inter-rater reliability for assessments of body height (ICC = 0.998), body mass (ICC = 0.998), and BMI (ICC = 0.988).

#### 2.3.2. Assessment of Shoulder Pain and Function

Shoulder impairment was assessed using the German version of the shoulder pain and disability index (SPADI) [17]. Separate pain and disability subscales as well as the total SPADI ranging from 0 (“no impairment”) to 100 (“worst impairment”) were calculated. The SPADI has been criticized for not addressing nocturnal pain, which has been found to have considerable influence on patients’ well-being [1]. Therefore, participants were also asked if they presently suffered from shoulder pain at night and whether they took any medication.

#### 2.3.3. Assessment of Shoulder Flexibility

Shoulder flexibility was assessed by the same skilled assessor during pre- and posttests using the neutral-zero method [18]. Active ROM was measured in a randomized order for the left and right shoulder joints in external and internal rotation at 90° abduction of the shoulder and elbow flexed at 90°. Starting from this position, participants were asked to perform a maximal external followed by a maximal internal rotation of the shoulder in the sagittal plane around the frontotransverse axis. Range of motion was measured to the nearest two degrees using a standardized analogue goniometer. On each side (i.e., left and right), two measurements were performed. A third was conducted if a difference larger than five degrees was observed between respective measurements.

#### 2.3.4. Assessment of Maximal Isometric Strength

**Figure 5:**
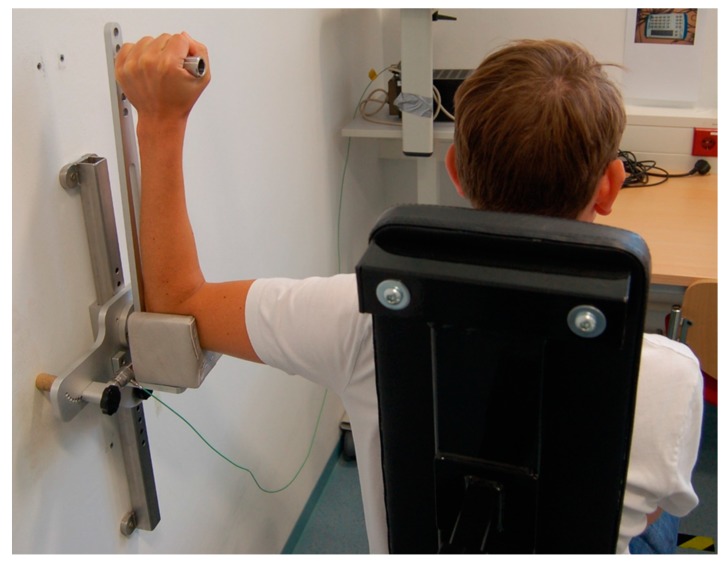
Assessment of maximal isometric muscle strength during maximal shoulder extension and flexion.

Maximal isometric strengths for the external and internal shoulder rotators of the left and right sides were measured with elbow flexed at 90° and shoulder abducted to 90° to the nearest 0.01 Nm using a force transducer (Kistler 9321B, Winterthur, Switzerland) and a custom-built dynamometer (Figure 5). Before measurements, the rotational axis of the dynamometer was aligned to participants’ shoulder joint position and the handlebar was adjusted to their respective forearm length. Individual adjustment of the dynamometer was noted for the posttesting. During measurements, participants were told to position their respective arm in the dynamometer, to grasp the handlebar, and to sit upright. Further, they were given a counterbalance (1.25 kg) to the opposite hand and were strapped to the chair. Participants were instructed to maximally externally or internally rotate their shoulder for three seconds. The start and end of each trial were verbally announced by the experimenter. Two maximal external and internal rotations with 30-seconds rest periods between trials were recorded for each side in a randomized order. A laptop with custom LabVIEW software (version 2016, National Instruments, Austin, TX, USA) was connected to the force transducer for data recording. The force signal was smoothed using a moving average filter with the window width set to 500 ms, and the best trial (i.e., highest value) per side and movement was used for analysis.

#### 2.3.5. Assessment of Strength Endurance

Strength endurance was tested using the closed kinetic chain upper extremity stability test (CKCUEST) and a metronome-paced strength endurance test. During the CKCUEST, female participants were instructed to take a kneeling push-up position with hands placed 36 inches (approx. 91.44 cm) apart, flat back, and shoulders perpendicular to the wrists. Male participants were asked to adopt the same position without supporting knees. Participants were then requested to alternately touch the supporting hand with the non-supporting hand as often as possible during three 15-s trials, separated by 45-s rest periods. In between trials, participants were allowed to relax in a comfortable position. The start and end of a trial were marked by an acoustic “beep”. Additionally, a 3-s verbal countdown was given before the start of a trial. The number of touches was recorded for each trial, and the best trial (i.e., maximal number of touches) was used for further analysis.

The movement performed during the metronome-paced strength endurance test was identical to the training exercise of the ALT-group. Participants were instructed to start with arms horizontal and to perform the maximal number of external rotations of the shoulder using the manual shoulder-training device and the rope. To standardize movement speed, participants were asked to reach the turning point (i.e., full external rotation and horizontal plane) in time with the metronome. The test was terminated when participants failed to perform a full external rotation and/or failed to follow the metronome for the second time. All participants performed the test with the metronome set at 30 bpm. The total number of reps was recorded and used for analysis.

### 2.4. Statistical Analyses

In order to obtain a medium-sized interaction effect, an a priori power analysis was performed using G* Power (version 3.1.9.2, Universität Kiel, Kiel, Germany) [19]. The following input parameters were used: effect size (*f* = 0.25), type I error (α = 0.05), type II error (β = 0.95), number of groups (*n* = 3), number of measurements (*n* = 2), and correlations between measurements (*r* = 0.60). Further, the dropout rate was estimated to reach 20%. In consideration of the aforementioned parameters, our analysis yielded a total sample size of 54 participants. Using the Shapiro–Wilk test (*p* > 0.05), data was analyzed for normal distribution. To test for significant differences between groups at pretesting a univariate analysis of variance (ANOVA) was used. Subsequently, a 2 (test: pre and post) × 3 (group: CON, TRA, and ALT) ANOVA with repeated measures on test was performed to detect differences between groups from pre- to posttest. Bonferroni-adjusted post hoc tests were carried out if significant test × group interaction effects occurred. Cohen’s *d* was calculated to determine the effect size with *d* = 0.2 representing small effects, *d* = 0.5 representing moderate effects, and *d* = 0.8 representing large effects. Statistical Package for Social Sciences version 24.0 was used for all statistical analyses with the significance level set at *p* < 0.05.

## 3. Results

Group mean values, standard deviations, percent change, and repeated-measure ANOVA results for all outcome variables are shown in Table 2. Generally, there were no statistically significant differences in pretesting values between the three groups. None of the participants reported any training or test-related injury.

### 3.1. Shoulder Pain and Function

The statistical analyses indicated significant main effects of Test as well as significant Test × Group interactions for the SPADI total scale, for the SPADI subscale “disability”, and for the SPADI subscale “pain” (Table 2). For both training groups, the post hoc analysis revealed improvements from pre- to posttesting that were larger for the ALT group (SPADI total scale: +51%, *p* < 0.001, *d* = 0.98; SPADI subscale “disability”: +58%, *p* < 0.001, *d* = 0.89; and SPADI subscale “pain”: +44%, *p* < 0.001, *d* = 0.91) compared to the TRA group (SPADI total scale: +28%, *p* = 0.002, *d* = 0.64; SPADI subscale “disability”: +24%, *p* = 0.004, *d* = 0.54; and SPADI subscale “pain”: +27%, *p* = 0.002, *d* = 0.75). For the CON group, we could not detect significant pre- to posttesting changes in any of the three measures of shoulder pain/function. Furthermore, the percentage of participants reporting nocturnal shoulder pain decreased in the ALT (−35%) but not in the TRA (+5%) and CON groups (±0%), whereas the proportion of participants taking anti-inflammatory drugs to counteract SIS symptoms was especially reduced in the ALT group (−24%), in the TRA group (−32%), and to a lesser extent in the CON group (−5%) from pre- to postintervention.

### 3.2. Shoulder Flexibility

For measures of shoulder flexibility, significant main effects of Test and significant Test × Group interactions were found for ROM external rotation of the left and right arms and for ROM internal rotation of the right arm (Table 2). In both training groups, the post hoc analysis indicated significant improvements from pre- to posttesting that were larger for the ALT group (ROM external rotation left arm: +28%, *p* < 0.001, *d* = 1.13; ROM internal rotation left arm: +55%, *p* = 0.002, *d* = 0.69; and ROM internal rotation right arm: +57%, *p* < 0.001, *d* = 1.00) compared to the TRA group (ROM external rotation left arm: +18%, *p* = 0.003, *d* = 0.75; ROM internal rotation left arm: +39%, *p* = 0.044, *d* = 0.48; and ROM internal rotation right arm: +41%, *p* = 0.014, *d* = 0.53). However, for ROM external rotation of the right arm, the ALT group (+17%, *p* = 0.003, *d* = 0.93) but not the TRA group (+6%, *p* = 0.207, *d* = 0.27) significantly enhanced their performance. No significant pre- to posttesting changes in any of the flexibility measures occurred for the CON group.

### 3.3. Maximal Isometric Strength

With regards to the maximal strength test, the statistical analyses revealed significant main effects of Test for MIS of the external and internal rotators of the left and right arm, yet a significant Test × Group interaction was found for MIS of the external rotators of the right arm only (Table 2). For the two training groups, the post hoc analysis showed improvements from pre- to posttesting for MIS of the external rotators of the right arm that were larger for the ALT (+54%, *p* < 0.001, *d* = 0.88) compared to the TRA group (+42%, *p* < 0.001, *d* = 0.46). No significant changes were found for the CON group from pre- to posttesting.

### 3.4. Strength Endurance

Concerning the strength endurance tests, our statistical analyses yielded significant main effects of Test and significant Test × Group interactions for the metronome-paced strength endurance test and for the CKCUEST (Table 2). In both training groups, the post hoc analysis showed enhancements from pre- to posttesting for the CKCUEST that were larger for the TRA (+19%, *p* = 0.001, *d* = 1.00) compared to the ALT group (+14%, *p* < 0.001, *d* = 0.66). However, for the metronome-paced strength endurance test, the ALT group (+89%, *p* = 0.012, *d* = 0.94) but not the TRA group (+23%, *p* = 0.665, *d* = 0.08) significantly improved their performance. Further, none of the strength endurance tests showed significant pre- to posttesting changes for the CON group.

## 4. Discussion

We investigated the effects of a TRA versus an ALT resistance exercise program. In this regard, we assessed proxies of shoulder pain/function (i.e., SPADI) and physical performance (i.e., shoulder flexibility, MIS, and strength endurance) in individuals with subacromial shoulder pain. Participants conducted a single exercise home-based strengthening training of the rotator-cuff muscles for eight weeks either with or without using a manual shoulder-training device during external rotation. The main findings of this study can be summarized as follows: a) eight weeks of training proved to be safe (i.e., no training or test-related injuries) and feasible; b) both training programs were effective and resulted in statistically significant enhancements in shoulder pain/function (i.e., SPADI) and physical performance (i.e., shoulder flexibility, MIS, and strength endurance); and c) the ALT group showed more frequent and larger improvements compared to the TRA group; specifically, ALT induced 10 significant enhancements (8 large and 2 medium sized effects) compared to 8 significant improvements (1 large, 5 medium, and 2 small sized effects) when using TRA.

### 4.1. Training Effects on Measures of Shoulder Pain and Function

In terms of shoulder pain/function, we can confirm our hypothesis that both exercise conditions will result in enhanced values but with larger improvements in the ALT as compared to the TRA group. More specifically, we observed improvements of 20.0 points in the ALT and 12.7 points in the TRA groups in total SPADI scores, whereas changes in the CON group were negligible (−0.3 points). According to Schmitt et al. [20], the standard error of measurement (SEM) reflecting a change which is most likely not explained by measurement error amounts to 7.75 points. Additionally, the minimal clinically important difference (MCID), indicating an actually meaningful change in function from the patient’s perspective amounts to 13.2 points for the total SPADI score [20]. Consequently, our results indicate that improvements in both training groups were beyond SEM. However, from the participant’s perspective, only conducting the ALT-training for eight weeks was effective to reduce perceived shoulder pain and disability during activities of daily living as improvements in total SPADI score in the ALT group (20.0 points) clearly exceeded the MCID. Improvements of the TRA training (12.7 points) missed the MCID value by merely 0.5 points. Therefore, the changes we observed in the TRA group may not necessarily indicate true improvement from a participant’s perspective, whereas the changes found in the ALT group seem to reflect clinically important change.

The SPADI improvements are similar to those reported in the literature following different means of conservative treatment (e.g., supervised training and PT). For example, in an observational study, Clausen et al. [21] found the total SPADI to improve by 23.0 points over a course of six months in patients with subacromial shoulder pain who received conservative treatment. However, therapy varied considerably between individuals and included repeated steroid injections, PT, and home-based exercises. In our study, conducting the single-exercise home-based elastic resistance training with the manual shoulder device induced similar effects in a markedly shorter period of time (8 weeks). Moreover, Littlewood et al. [11] compared the effects of a single-exercise home-based strengthening training of the rotator-cuff to traditional PT in patients with rotator-cuff tendinopathy over a one year period. After three months of intervention, total SPADI was reduced by 12.4 points in the group conducting the home-based training and by 16.7 points in the traditional PT group. Improvement in the home-based training group is similar to the improvement we observed in the TRA group after eight weeks. However, both training regimens in the study by Littlewood et al. [11] failed to improve total SPADI scores beyond measurement error during the first three months of intervention, whereas we detected a meaningful change in the group training with the shoulder device after eight weeks. Nevertheless, Littlewood and colleagues [11] observed meaningful changes (>18 points) for both intervention groups after six and twelve months. Similar results were obtained by Kromer et al. [22], who compared the effects of a combined PT and individualized exercise program to an individualized exercise program alone in people with subacromial shoulder pain, with statistically significant changes below the MDC after five weeks but further improvements exceeding MDC after 12 weeks of intervention.

Further, approximately 82% of the participants in our study suffered from nocturnal shoulder pain at pretest, which corresponds to the percentage (88%) reported by Ostor et al. [1]. However, at posttest, we observed that, compared to the TRA and CON groups, only the ALT training was effective to reduce nocturnal shoulder pain. More specifically, the proportion of participants suffering from shoulder pain at night had decreased by more than one third after subjects trained with the shoulder device for eight weeks whereas little to no change was observed in the other two groups. Unfortunately, we did not perform any measurements during sleep, leaving usable underlying mechanisms, although it might be speculated that reduced activity of, for example, the deltoid muscle may play a role. Nevertheless, we also cannot rule out that this finding may, at least to some degree, be associated with a placebo effect. As nocturnal shoulder pain is considered one of the major contributors to impaired quality of life in patients with subacromial shoulder pain, this finding highlights the superiority of the ALT training over the TRA and the CON regimen. Consequently, the ALT as well as the TRA training proved to be suitable and effective in order to improve measures of self-reported shoulder pain and function. However, the ALT training using the manual shoulder-training device produced larger improvements, especially with respect to nocturnal shoulder pain.

### 4.2. Training Effects on Measures of Physical Performance

The hypothesis that both exercise conditions will result in improved physical performance and that the ALT group shows larger enhancements as compared to the TRA group was correct for most of the investigated variables. This is largely in accordance with previous studies [23,24,25] investigating the effects of conservative treatments on measures of shoulder function in patients with subacromial shoulder pain. For example, Bae et al. [23] observed statistically significant increases in active ROM during internal (+19%) and external (+17%) rotation of the shoulder in patients with subacromial shoulder pain after participants conducted a combination of PT, strengthening exercises, and motor control training three times a week for four weeks. Lombardi et al. [25] found slightly smaller increases in ROM during internal (+13%) and external (+9%) rotation of the shoulder in people with subacromial shoulder pain who participated in an eight-week, supervised, progressive resistance training for the shoulder muscles. However, these increases were not statistically significant when compared to changes observed in a matched control group. Compared to these two studies, we detected similar improvements concerning internal (left arm: +39%, right arm: +41%) and smaller improvements during external (left arm: +18%, right arm: +6%) rotation in the TRA group. However, the ALT training resulted in considerably larger improvements in active ROM especially during internal (left arm: +55%, right arm: +57%) but also in external (left arm: +28%, right arm: +17%) rotation of the shoulder joint. For people with subacromial shoulder pain, such an advantage in flexibility probably results in higher quality of life as tasks of everyday living (e.g., dressing) become much more accomplishable or accomplishable at all. It may be speculated that the ALT training using the shoulder-training device may enhance intramuscular control by reducing deltoid muscle activity during external rotation [14], thus allowing more motion of the shoulder joint. Consequently, conducting the ALT training using the manual shoulder device is recommended over the TRA training to increase active ROM during external and internal shoulder rotation in patients with subacromial shoulder pain.

Given that there is no study available which used the same custom-built dynamometer to measure MIS of the external and internal rotators of the shoulder, our findings have to be compared with results originating from studies using similar testing equipment. For example, Bang and Deyle [24] measured MIS of internal and external rotators in supine position using an Accuforce II electric dynamometer and investigated the effects of three weeks of exercise therapy to those of a combination of exercise and manual therapy in adults with subacromial shoulder pain. In their study, combining exercise therapy with manual therapy resulted in statistically significant increases in MIS of the external (+30%) and internal (+14%) rotators, whereas little changes (external: +2%, internal: +4%) were observed following exercise therapy only. This is in contrast to our results as we observed statistically significant increases in MIS of external/internal rotators of patients with subacromial shoulder pain after conducting a single-exercise home-based training using Therabands^®^ (TRA group; external: +26%–32%, internal: +16%–22%) or the shoulder device (ALT group; external: +26%–42%, internal: +17%–32%). Even though TRA and ALT training represent simple exercise therapy, they resulted in similar (i.e., TRA) or even larger (i.e., ALT) strength gains compared to the combination of exercise and manual therapy in the study by Bang and Deyle [24]. However, these differences may be a consequence of different intervention periods. Participants in our study trained for eight weeks, whereas the training regimen lasted only three weeks in the study of Bang and Deyle [24].

In another study, Bae et al. [23] observed larger increases (+44%) than we did (+26%–42%) in external rotator strength following four weeks of resistance training combined with motor control therapy, but they reported only small albeit statistically significant gains in internal rotator strength (+3%). However, these researchers assessed isokinetic muscle strength at 60°/s, which may limit the comparability of those findings and our results. In comparison to the studies discussed, TRA and especially ALT training regimens were not only effective to improve MIS of external but, to a great extent, also internal shoulder rotators. Finally, the observed increases in MIS (TRA group: +16%–32%; ALT group: +17%–42%) represent actual change as they clearly exceeded the 10% margin of clinical significance [26]. Therefore, the ALT is recommended over the TRA to increase MIS of external/internal shoulder rotators in adults with subacromial shoulder pain.

With respect to strength endurance, we observed statistically significant improvements in the metronome-paced test with the shoulder device for the ALT group and statistically significant enhancements in the CKCUEST for both training groups. To the best of our knowledge, no other interventional study with patients suffering from subacromial shoulder pain applied these tests; thus, a classification of our results is difficult. However, Tucci et al. [27] reported the mean number of touches during the CKCUEST for middle-aged patients with subacromial shoulder pain. With an average of 11.9 touches, the participants in that study performed considerably worse than those in our study who achieved 16.5–18.3 touches at preintervention, indicating that shoulder impairment may have been more pronounced in participants of the study by Tucci et al. [27]. Moreover, a change of 2.7 touches in the CKCUEST is deemed to reflect actual change in subacromial shoulder pain patients (22), which was almost achieved in the ALT group (+2.6 touches) and obtained in the TRA group (+3.0 touches) following intervention. Although the TRA (+18%) was slightly more effective than the ALT (+14%) with regard to the CKCUEST, the ALT (+55%) compared to the TRA (+4%) was clearly more effective concerning the metronome-paced strength endurance test. Consequently, both trainings were effective to improve strength endurance of the external rotators of the shoulder.

Altogether, our results are in accordance with those of a systematic review of randomized controlled trials [28] which showed that conservative treatments and especially strengthening exercises of the shoulder muscles are effective means not only to reduce self-reported shoulder pain and function but also to improve muscular strength as well as ROM of the shoulder joint in patients suffering from subacromial shoulder pain. However, the vast majority of studies included in that review used a multi-exercise approach, often combining strengthening exercises with other means of conservative treatment (e.g., manual therapy and taping) causing interventions to be time-consuming and leaving patients reliant on supervision through experts (e.g., physical therapists). Conducting a single exercise, unsupervised, home-based training either with or without the shoulder-training device resulted in large and mostly significant improvements in active ROM, MIS, and strength endurance in adults with subacromial shoulder pain. However, we observed larger and more frequent effects for the ALT as compared to the TRA group, potentially indicating an advantage of the ALT training.

### 4.3. Limitations

There are a few limitations with this study that need to be addressed. First, our conclusions only apply to the population investigated (i.e., middle-aged adults with subacromial shoulder pain) and the training modalities (i.e., duration, frequency, and number of sets/reps) we adopted for this study. Consequently, the effectiveness of the respective training regimens in other age groups (e.g., young and older adults) and/or clinical populations (e.g., frozen shoulder and post-surgery) remains unclear. Moreover, we did not conduct any follow-up assessment and therefore cannot comment on long-term (>8 weeks) and/or attenuation effects of the applied training regimens. However, improvements in shoulder pain and physical performance following the applied training regimes may also be attributed to structural changes (e.g., tendon), especially in the long run. Therefore, future studies should use a longer intervention period (e.g., ≥16 weeks) and can include imaging techniques (e.g., ultrasound and magnetic resonance imaging) to examine structural changes following the training. Another limitation relates to the assessment of MIS of the shoulder rotators which was measured with the shoulder abducted to 90°. This position is similar to the position during the ALT training, whereas the TRA group trained with 0° abduction. Therefore, additionally measuring MIS of the shoulder rotators with 0° abduction would have been good. Similarly, the exercise of the metronome-paced strength endurance test mimicked the training exercise of the ALT group, thus potentially biasing our results in favor of the ALT group. Due to the scheduling of the study, assessors were only blinded with respect to the two intervention groups (i.e., TRA and ALT) but not regarding the CON group. Especially when testing a new training device, results may be influenced by a placebo effect as participants may experience improvements simply due to their expectations. However, we tried to control this by using valid and reliable measures of shoulder pain/function and physical performance. Finally, although we tried to match elastic bands of the training groups with respect to their resistance, this was not completely possible as both groups used different products.

## 5. Conclusions

The present study investigated the effects of a TRA versus an ALT strengthening exercise program on measures of shoulder pain/function and physical performance in individuals with subacromial shoulder pain. Both training programs proved to be safe and feasible. Most importantly, eight weeks of a single-exercise home-based training either with or without the manual shoulder device resulted in improvements in self-reported shoulder pain and function, shoulder flexibility, MIS, and strength endurance. For most of the investigated variables, significant improvements were more frequent and larger in the ALT group using the manual shoulder-training device Schulterhilfe^®^ compared to the TRA group. Therefore, the single-exercise home-based elastic resistance strengthening program using the shoulder-facilitating device seems to represent a suitable alternative to traditional training regimes for patients with subacromial shoulder pain to improve shoulder function and physical performance.

## Figures and Tables

**Table 1:** Descriptive data by group.

Characteristic	CON (*n* = 20)	TRA (*n* = 19)	ALT (*n* = 17)	*p*-Value
Sex (f/m)	11/9	10/9	8/9	-
Age (years)	53.0 ± 6.6	54.3 ± 8.4	52.7 ± 7.0	0.771
Body height (cm)	170.4 ± 9.8	174.7 ± 9.7	171.1 ± 8.5	0.319
Body mass (kg)	82.0 ± 18.0	83.0 ± 17.9	79.6 ± 26.6	0.886
BMI (kg/m^2^)	28.0 ± 4.2	27.2 ± 5.4	26.9 ± 7.1	0.823

Values are presented as mean ± standard deviation. ALT = alternative training group using elastic bands in combination with the manual shoulder-training device Schulterhilfe^®^; BMI = body mass index; CON = passive control group; f = female; m = male; TRA = traditional training group using elastic bands (Theraband^®^) only.

**Table 2:** Outcomes of the univariate analysis of variance with repeated measures.

Outcome	CON (*n* = 20)			TRA (*n* = 19)			ALT (*n* = 17)			*p*-/*d*-Value	
	Pre	Post	∆% *	Pre	Post	∆% *	Pre	Post	∆% *	T	T × G
*Shoulder pain/function*											
SPADI total scale (pt)	41.0 ± 19.8	40.7 ± 21.9	+4 (43)	44.1 ± 19.2	31.4 ± 20.5	+28 (36)	40.0 ± 21.7	20.0 ± 16.3	+51 (34)	<0.001 (1.68)	<0.001 ^#^ (1.25)
SPADI subscale “disability” (pt)	36.5 ± 21.9	35.2 ± 22.2	+5 (87)	37.3 ± 20.3	26.3 ± 20.7	+24 (55)	35.4 ± 22.4	15.8 ± 13.0	+58 (33)	<0.001 (1.56)	0.001 ^#^ (1.09)
SPADI subscale “pain” (pt)	48.1 ± 18.8	49.5 ± 23.1	−1 (34)	55.1 ± 19.3	39.6 ± 21.6	+27 (32)	47.5 ± 21.9	26.8 ± 23.3	+44 (39)	<0.001 (1.48]	<0.001 ^#^ (1.22)
*Shoulder flexibility*											
ROM, external rotation, left arm (°)	67.9 ± 22.0	66.8 ± 21.5	−1 (18)	72.5 ± 14.5	83.7 ± 15.3	+18 (24)	73.1 ± 15.7	91.8 ± 17.1	+28 (23)	<0.001 (1.57)	<0.001 ^#^ (1.34)
ROM external rotation, right arm (°)	83.0 ± 15.0	80.8 ± 14.8	−2 (14)	82.0 ± 12.8	85.9 ± 15.3	+6 (19)	82.5 ± 13.1	94.9 ± 13.7	+17 (20	0.011 (0.73)	0.006 ^#^ (0.92)
ROM, internal rotation, left arm (°)	50.3 ± 17.5	51.4 ± 15.7	+21 (77)	47.9 ± 20.1	58.4 ± 23.5	+39 (69)	51.2 ± 21.6	66.6 ± 22.7	+55 (80)	<0.001 (1.02)	0.057 ^#^ (0.67)
ROM, internal rotation, right arm (°)	48.3 ± 16.7	46.4 ± 17.4	−1 (30)	45.4 ± 21.5	56.4 ± 19.9	+41 (36)	48.0 ± 12.5	66.2 ± 20.3	+57 (100)	<0.001 (1.20)	0.001 ^#^ (1.10)
*Maximal strength*											
MIS external rotators, left arm (Nm)	9.3 ± 4.7	11.3 ± 5.8	+31 (48)	13.3 ± 6.5	16.7 ± 8.2	+37 (50)	11.4 ± 5.3	14.4 ± 5.4	+39 (37)	<0.001 (1.49)	0.510 (0.33)
MIS external rotators, right arm (Nm)	12.3 ± 7.3	13.6 ± 6.1	+36 (67)	13.1 ± 7.2	17.3 ± 8.9	+42 (49)	11.3 ± 5.1	16.1 ± 5.6	+54 (53)	<0.001 (1.90)	0.010 ^#^ (0.87)
MIS internal rotators, left arm (Nm)	20.7 ± 15.1	20.9 ± 14.5	+12 (36)	23.2 ± 16.4	26.8 ± 17.7	+26 (38)	19.7 ± 12.3	23.1 ± 11.9	+35 (59)	0.012 (0.75)	0.225 (0.35)
MIS internal rotators, right arm (Nm)	22.0 ± 16.6	22.1 ± 15.1	+27 (72)	24.0 ± 18.6	29.3 ± 19.4	+41 (64)	17.8 ± 8.6	23.5 ± 10.8	+35 (39)	0.001 (1.00)	0.217 (0.35)
*Strength endurance*											
Metronome-paced strength endurance test (*n*)	52.3 ± 66.5	56.1 ± 95.3	+1 (39)	37.0 ± 20.6	38.5 ± 13.7	+23 (66)	42.8 ± 18.0	66.4 ± 29.3	+89 (141)	0.015 (0.70)	0.051 ^#^ (0.70)
CKCUEST (*n*)	17.2 ± 5.2	18.6 ± 5.4	+10 (16)	16.5 ± 3.0	19.5 ± 2.9	+19 (13)	18.3 ± 3.0	20.9 ± 3.7	+14 (8)	<0.001 (2.77)	0.017 ^#^ (0.83)

Values are mean values ± standard deviations. * The mean of the individual percentage changes with the standard deviation in parentheses, whereas a positive/negative value indicates improvement/decrement. ^#^ The post hoc analysis showed statistically significant differences between groups. Figures in brackets are effect sizes (Cohen’s *d*) with 0 ≤ *d* ≤ 0.49 indicating small, 0.50 ≤ *d* ≤ 0.79 medium, and *d* ≥ 0.80 large effects. ALT = alternative training group using elastic bands in combination with the manual shoulder-training device Schulterhilfe^®^; CKCUEST = closed kinetic chain upper extremity stability test; CON = passive control group; G = between-subject factor “group”; MIS = maximal isometric strength; ROM = range of motion; T = within-subject factor “test”; TRA = traditional training group using elastic bands (Theraband^®^) only.
